# Women’s health in the Lund area (WHILA) - Alcohol consumption and all-cause mortality among women – a 17 year follow-up study

**DOI:** 10.1186/s12889-016-2700-2

**Published:** 2016-01-12

**Authors:** Patrik Midlöv, Susanna Calling, Ashfaque A. Memon, Jan Sundquist, Kristina Sundquist, Sven-Erik Johansson

**Affiliations:** Center for Primary Health Care Research, Department of Clinical Sciences, Lund University, Jan Waldenströms gata 35, 205 02 Malmö, Sweden

**Keywords:** Alcohol, Mortality, Women, J-shaped

## Abstract

**Background:**

Alcohol consumption contributes to many negative health consequences and is a risk factor for death. Some previous studies however suggest a J-shaped relationship between the level of alcohol consumption and all-cause mortality. These findings have in part been suggested to be due to confounders. The aim of our study was to analyze the relationship between self-reported alcohol intake and all-cause mortality in women, adjusted for sociodemographic, lifestyle factors and diseases such as diabetes and previous ischemic heart disease.

**Methods:**

All women aged 50–59 years (born between 1935 and 1945) that lived in any of the five municipalities in southern Sweden were invited to participate in a health survey. From December 1995 to February 2000 a total of 6916 women (out of 10,766, the total population of women in 1995) underwent a physical examination and answered a questionnaire. We followed the women from the day of screening until death, or if no event occurred until May 31st 2015. Mortality was ascertained through the national cause-of-death register.

**Results:**

In this study a total of 6353 women were included. Alcohol consumption showed a J-formed relationship with mortality, when adjusted for education, marital status, smoking, BMI, physical fitness, diabetes and ischemic heart disease before screening. Non consumption of alcohol was associated with increased mortality as well as higher levels of consumption, from 12 grams per day and upwards.

**Conclusions:**

There was a clear J-shaped relation between the amount of alcohol consumption and all-cause mortality even after controlling for sociodemography, lifestyle factors and diseases such as diabetes and previous ischemic heart disease. The observed protective effect of light drinking (1–12 grams/day) could thus not be attributed to any of these known confounders.

## Background

Alcohol consumption contributes to a wide range of negative acute and chronic health consequences and is one of the major factors for burden of disease [1]. The net burden caused by alcohol consumption in the European Union in recent years has been estimated to be one in seven deaths in males and one in 14 deaths in females [2]. There are different mechanisms by which alcohol affects the risk of death. Changes in lipoproteins such as increasing HDL cholesterol and apolipoprotein A1, as well as an association between alcohol intake and a favourable haemostatic profile have been suggested [3]. Another possible mechanism is the U-shaped association between alcohol consumption and fibrinogen concentrations [4].

Alcohol consumption does not only increase the risk for many diseases but is also a risk factor for death by external causes i.e. injuries [5]. In Sweden, alcohol abuse increases the relative risk of death by accidents by 14.9 in women and by 9.1 in men [6].

There are however disparities in the connection between mortality and the use of alcohol. Previous studies have suggested a J-shaped or even U-shaped relationship between the level of alcohol consumption and all-cause mortality [7, 8]. The increased risk for abstainers has been due to a greater possibility of cardiovascular mortality [9, 10] including stroke [11]. However, other studies have questioned the J-shaped relationship between the level of alcohol consumption and all-cause mortality and suggested it to be attributable to weak adjustment for confounders or misclassification bias due to mixing former drinkers with lifelong abstainers [12]. Possible confounding factors such as marital status [13], education [14], physical activity [15] and obesity [16] may affect these associations. Diabetes could be another potential confounding factor since moderate alcohol consumption has been reported to be inversely associated with the risk of diabetes, whereas high alcohol consumption could be associated with a higher risk [17] especially in women [18]. It has been suggested that women have an increased risk for all-cause mortality due to alcohol consumption compared with men [19]. There is a need for more studies on women’s alcohol consumption and effect on mortality.

In a previous study from the same population, which had a shorter follow-up time, (9 years instead of 17 years) alcohol abstainers had no increased mortality after adjusting for socio-demographic and health predictors [20].

The main aim of the present study was to analyze the relationship between self-reported alcohol intake and all-cause mortality in women, also adjusted for sociodemographic, lifestyle factors and diseases such as diabetes and previous ischemic heart disease.

## Method

Data for the present study were retrieved from the Women’s Health in Lund Area (WHILA) study [21]. All women aged 50–59 years (born between 1935 and 1945) that lived in any of the five municipalities in southern Sweden were invited to participate in a health survey. From December 1995 to February 2000 a total of 6916 women (out of 10,766, the total population of women in 1995) underwent a physical examination and answered a questionnaire. Thus, the participation rate was 64.2 %. The questionnaire that was distributed to all participants has been described in a previous Swedish study from 2002 [21]. After providing written consent the participants spent up to 2 h to answer the questionnaire, which included questions about alcohol consumption, sociodemographic data and health. If the participants had any queries they could ask a nurse. There was no financial reimbursement for participation.

We followed the women from the day of screening until death, or if no event occurred until May 31st 2015. Mortality was ascertained through the national cause-of-death register.

### Outcome variables

*All-cause mortality*: All deaths recorded from baseline until end of the follow-up on May 31st 2015. In total there were 579 deaths and on average 17 years follow-up.

### Explanatory variables

All explanatory variables, except age, weight and height, ischemic heart disease (IHD), and diabetes were self-reported in the questionnaire. Age was taken from the population register, while weight, height, and diabetes were obtained from a clinical investigation, while IHD was taken from the Swedish National Patient Registry.

*Age,* age at screening, Age centred round its mean (56 years).

*Education;* Educational level was categorized into low (≤12 years) and middle/high (>12 years of schooling).

*Partnership status was dichotomized into married vs. single living/divorced/widow.*

*Body Mass Index (BMI;* (weight (kg)/height^2^ (m)) was categorized into underweight (<18.5), normal weight (18.5–24.9), overweight (25–29.9) and obese (≥ 30).

*Alcohol consumption* (grams of alcohol per day) comprised of three groups: none, 0.1–11.9, and ≥12.0 (12 grams of pure alcohol equals one drink). Alcohol consumption was assessed by the following question: ‘How much alcohol do you drink in an ordinary week?’ and to report the quantity of glass/bottles (specified in centilitres) of beer, wine and spirits respectively, or the option ‘no alcohol’. Each participant’s total consumption of alcohol was converted into grams of alcohol for beer, wine and spirits separately and then divided into total grams of alcohol per day. Non-drinkers were identified by the question: Do you on the whole drink any alcohol: Yes/No, where No is classified as non-drinkers.

The women were divided into three groups:

- 0 g Women who did not drink any alcohol in an ordinary week.
- 0.1-11.9 g alcohol per day.
- ≥12 g alcohol per day.

The variable is also treated as continuous in the models. We used restricted cubic splines in order to show the hazard ratios over the entire scale of alcohol consumption.

*Physical fitness* (based on a 7-level ordinal scale ranging from very poor to very good) was dichotomized: Poor (1–3) and Middle/Good (4–7).

*Ischemic heart disease (IHD)* before screening was dichotomized (obtained from the Swedish National Patient Registry diagnoses: 410–414/I20–I24): Yes/No.

*Diabetes* (obtained from the clinical investigation): Yes/No

### Statistical method

In order to compensate for missing we weighted data by N_i_/n_responders_ per 1-year age-group (50–59). The response rate varied in the different age-groups between 58.9 % (youngest) and 66.7 % (oldest), on average 64.2 %.

We used Cox proportional hazard model to analyze the relationship between alcohol consumption and all-cause mortality, adjusted for potential confounders. We had no specific hypotheses for interactions, therefore we tested the first order interactions for all variables except diabetes and ischemic heart disease, in total 21 interactions vs. alpha = (0.05/21) = 0.0024.

To show the hazard ratios of alcohol consumption over the entire scale we used restricted cubic splines by including alcohol as a continuous variable in the models.

### Ethics

This study was approved by the ethics committee in Lund (approval no. 174–95 and 2011/494). Participation in the study was based on informed consent.

## Results

The participants were on average 56 years of age at the screening. In this study only 6353 women were included, mostly due to missing in alcohol consumption. A total of 563 women were excluded (Table 1).

**Table 1:** Missing in the different variables

Variable	Missing (excluded)	Response
Age	0	6916
Education	125	
Marital status	21	
BMI	0	6916
Alcohol intake	288	
Fitness	58	
Smoking	134	
Diabetes	67	
Ischemic heart disease	0	6916
Time	0	6916
Totals	563	6353

**Fig. 1:**
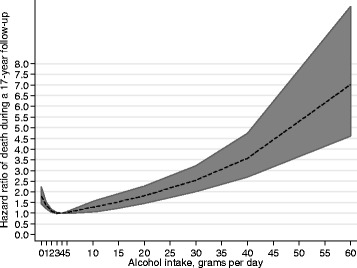
The Hazard ratio of all-cause mortality by alcohol consumption (grams per day), adjusted for age

**Fig. 2:**
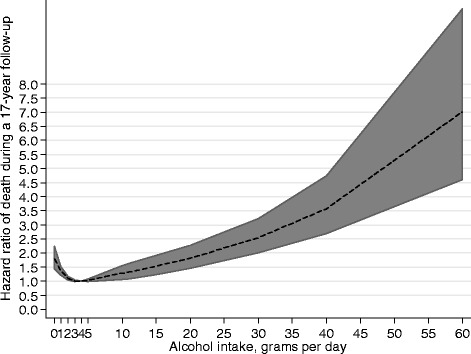
The Hazard ratio of all-cause mortality by alcohol consumption (grams per day), adjusted for age, education, marital status, smoking, BMI, fitness, diabetes, and ischemic disease before baseline

Alcohol consumption showed a J-formed relationship with mortality, when adjusted for age (Fig. 1). This relationship remained after adjusting for education, marital status, smoking, BMI, physical fitness, diabetes, and ischemic heart disease before screening (Fig. 2). There were fewer participants with middle/high educational level and more non-smokers among non-drinkers (Table 2). Diabetes and obesity were also more common among non-drinkers (Table 2).

**Table 2:** The distribution (%) of the variables *n* = 6 353; number of deaths = 579; follow-up on average 17 years. Test of distribution by chi-2

		Alcohol consumption (grams/day)			
		0	0.1–11.9	≥12	All
Variable	Level	% (*n* = 1639)	% (*n* = 3880)	% (*n* = 834)	% (*n* = 6353)
Total		25.8	61.1	13.1	100
Age (mean)	Continuous	56.9	56.3	55.7	56.4
Education					
	Low	37.8	20.2	13.0^*)^	23.8
	Middle/High	62.2	79.8	87.0	76.2
Partnership status					
	Married	65.9	75.1	71.9^*^	72.3
	Single/divorced/widow	34.1	24.9	28.1	27.7
Fitness					
	Poor	23.3	16.1	16.5^*^	18.0
	Middle/Good	76.7	83.9	83.5	82.0
Smoking					
	Non-smoker	64.1	60.1	43.7^*)^	59.0
	Former smoker	13.9	21.9	27.6	20.5
	Daily smoker	22.0	18.0	28.7	20.5
BMI					
	Underweight	1.5	1.0	1.2^*^	1.2
	Normal	39.7	53.6	57.4	50.5
	Overweight	36.8	34.0	32.6	34.5
	Obese	22.0	11.4	8.9	13.8
Diabetes					
	Yes	3.8	1.9	1.3^*^	2.3
	No	96.2	98.1	98.7	97.7
Ischemic heart disease (before screening)	Yes	2.1	1.5	0.7^**^	1.5
	No	97.9	98.5	99.3	98.5

^*^ *p* < 0.0001;^**^ *p* < 0.05

Non consumption of alcohol was associated with increased mortality, as well as higher levels of consumption, from 12 grams per day and upwards.

Poor education, single marital status, smoking, obesity, poor physical fitness, diabetes, and ischemic heart disease were all associated with higher mortality (Table 3).

**Table 3:** All-cause mortality rates per 10,000 person-years at risk with 95 % confidence interval and Hazard Ratios for all-cause mortality adjusted for age; *n* = 6 353; number of deaths = 579; follow-up on average 17 years

		All-cause mortality			
Variable	Level	Rate	95 % CI	HR	95 % CI
Education					
	Low	75	65–87	1.50	1.22–1.79
	Middle/High	47	43–52	1	Ref
Partnership status					
	Married	47	43–52	1	Ref
	Single/divorced/widow	70	62–81	1.50	1.26–1.77
Fitness					
	Poor	77	66–91	1.65	1.37–1.99
	Middle/Good	49	44–54	1	Ref
Smoking					
	Non-smoker	43	38–49	1	Ref
	Former smoker	50	42–61	1.20	0.97–1.50
	Daily smoker	88	77–101	2.18	1.81–2.62
BMI					
	Underweight	150	98–241	3.41	2.11–5.51
	Normal	47	42–54	1	Ref
	Overweight	52	46–60	1.06	0.88–1.27
	Obese	73	60–88	1.46	1.16–1.83
Alcohol consumption (g/day)					
	None	73	64–84	1.61	1.34–1.92
	0.1–11.9	36	31–41	1	Ref
	≥12	65	53–80	1.59	1.25–2.01
Diabetes					
	Yes	123	81–197	2.16	1.47–3.17
	No	53	48–57	1	Ref
Ischemic heart disease (before screening)	Yes	128	82–210	2.15	1.36–3.41
	No	43	39–47	1	Ref

For example, women who did not consume alcohol had a similar age-adjusted hazard ratio of all-cause mortality as women with a high consumption of alcohol; HR 1.61 (1.34–1.92) and 1.59 (1.25–2.01) respectively. Socioeconomic factors, such as low educational status or living alone, had similar increased risks of all-cause mortality with hazard ratios of 1.50 and 1.50 respectively, adjusted for age. Women that were underweight had the highest hazard ratio of all-cause mortality; 3.41 (2.11–5.51).

In Table 4 we adjusted for all included variables. In this model we categorized alcohol consumption and lost quite a lot of information compared with the figure where the model was based on continuous alcohol consumption. The loss of information was especially large among heavy drinkers. The hazard ratio for women with no alcohol consumption decreased from 1.61 to 1.36. For women with high alcohol consumption the risk decreased from 1.59 to 1.49 after adjusting for education, marital status, fitness, smoking, BMI, diabetes, and ischemic heart disease. All other hazard ratios decreased but remained significant, with the exception of obesity as that was no longer significant.

**Table 4:** All-cause mortality hazard ratios (HR) with 95 % confidence interval and Hazard Ratios for all-cause mortality; *n* = 6 353; number of deaths = 579; follow-up on average 17 years adjusted for all other variables

All-cause mortality			
Variable	Level	HR	95 % CI
Agec (age-56)	Continuous	1.11	1.08–1.14
Education			
	Low	1.35	1.12–1.62
	Middle/High	1 (ref)	
Partnership status			
	Married	1 (ref)	
	Single/divorced/widow	1.32	1.11–1.57
Fitness			
	Poor	1.46	1.20–1.78
	Middle/Good	1 (Ref)	
Smoking			
	Non-smoker	1 (ref)	
	Former smoker	1.18	0.94–1.47
	Daily smoker	1.91	1.58–2.32
BMI			
	Underweight	2.68	1.64–4.37
	Normal	1 (ref)	
	Overweight	1.02	0.84–1.23
	Obese	1.22	0.95–1.56
Alcohol consumption			
	None	1.36	1.13–1.64
	0.1–11.9 g/day	1 (ref)	
	≥12 g/day	1.49	1.18–1.89
Diabetes			
	Yes	1.78	1.19–2.70
	No	1 (ref)	
Ischemic heart disease (before screening)	Yes	1.77	1.10–2.84
	No	1 (ref)	

### Interactions

There were no significant interactions in the all-cause mortality model.

## Discussion

In this prospective cohort study of middle-aged women, with over 17 years of follow-up, there was a clear J-shaped relation between amount of alcohol consumption and all-cause mortality even after controlling for sociodemography, lifestyle factors and diseases such as diabetes and previous ischemic heart disease. The observed protective effect of light drinking (1–12 grams/day) could thus not be attributed to any of these known confounders.

The J-shaped mortality curve observed in this study among light to moderate drinkers is consistent with previous studies [1, 7]. The reasons are not clear but there remains the possibility that the consumption of small amounts of alcohol may protect against the development of cardiovascular disease [10, 22, 23]. This might be due to the effects of alcohol on cardiovascular biomarkers e.g. high-density lipoprotein cholesterol, adiponectin, fibrinogen and the effect on haemostatic factors [24, 25].

It has also been shown that moderate alcohol consumption is associated with a lower risk of mortality and diabetes [26]. One plausible mechanism that might explain this association is the enhanced insulin sensitivity in light-to-moderate alcohol consumers [27], but the reasons behind this remain unclear. It is also worth mentioning, although we could not control for this, that the lower mortality risk in moderate drinkers could be confounded by better developed social networks found among moderate drinkers.

High educational level was least common among abstainers. This association has been reported previously [28]. The association between educational level and mortality might be due to health behaviours [29, 30]. The association between alcohol consumption and BMI was weak in our study. This is in line with previous results, which indicated that the precise effect of alcohol on body weight remains to be determined [31].

The findings in our study are especially important since women might have an increased risk of alcohol-related mortality compared with men [32]. There is a need for interventions to decrease alcohol consumption among women with moderate to heavy alcohol intake.

A discussion of alcohol consumption should form a part of preventive counselling but all possible effects of alcohol use must be considered. The amount of alcohol intake where the protective effects exceed the adverse effects is unclear. Alcohol drinking is associated with many health problems. Increased consumption of alcohol should not be recommended as a prevention of mortality (or cardiovascular disease). Any advice regarding the consumption of alcohol should be adjusted to factor in the risks and potential benefits for each individual patient. One should bear in mind that there is no control mechanism for alcohol purchase as e.g. for prescription of drugs. Alcohol is available and when recommending to increasing alcohol intake there is a risk for consumption levels that increases mortality.

### Strengths and limitations

The strengths of the present study are the prospective design and the large sample of women drawn from the general population. We have a long follow-up period and the data has been adjusted for several possible confounders. By using restricted cubic splines we showed the hazard ratios of the entire scale of alcohol consumption with adjustment for all other variables, without any loss of information.

Epidemiological studies have obvious limitations and the results must be interpreted cautiously. Abstainers may differ from drinkers in aspects that we have not adjusted our model for. There might be confounders that we are not aware of. In addition, some of the abstainers may in fact not be complete teetotallers. In this study we could not separate participants who used alcohol on a less than weekly from those who were total abstainers.

Another problem of using baseline questionnaires in follow-up studies is that alcohol consumption may change over time. We assume that the level of alcohol consumption at baseline is an accurate estimate of exposure throughout several years. In our study it is not possible to differentiate between former drinkers and lifelong abstainers.

There might be a bias in the self-reporting of alcohol consumption [33, 34]. The possibility of higher rate of under-reporting of consumption in heavy drinkers cannot be ruled out. Self-reported data can be influenced by several factors, including social context and cultural norms, people may alter their response according to perceived social desirability [35]. It has however been shown that self-reported alcohol consumption can be reliable [36].

There is also a possibility that non-responders are different from the responders and non-responders may have an increased hazard ratio of alcohol-related mortality [37].

When it comes to IHD and diabetes we cannot rule out the possibility that these diseases may have occurred after inclusion in the study. In that case we do not know about these diagnoses, we only have data at baseline.

## Conclusion

In conclusion, no alcohol consumption or alcohol consumption of more than 12 g per day (seven drinks per week) were associated with increased mortality in women. This study underlines the need for further studies regarding mechanisms of the effect of alcohol on mortality.
